# Osteoarchaeological Studies of Human Systemic Stress of Early Urbanization in Late Shang at Anyang, China

**DOI:** 10.1371/journal.pone.0151854

**Published:** 2016-04-06

**Authors:** Hua Zhang, Deborah C. Merrett, Zhichun Jing, Jigen Tang, Yuling He, Hongbin Yue, Zhanwei Yue, Dongya Y. Yang

**Affiliations:** 1 Department of Archaeology, Simon Fraser University, Burnaby, British Columbia, Canada; 2 Department of Anthropology, University of British Columbia, Vancouver, British Columbia, Canada; 3 Institute of Archaeology, Chinese Academy of Social Sciences, Beijing, China; 4 SFU-JLU Joint Centre for Bioarchaeological Research, Department of Archaeology, Simon Fraser University, Burnaby, British Columbia, Canada; University of Otago, NEW ZEALAND

## Abstract

Through the analysis of human skeletal remains and mortuary practice in Yinxu, this study investigates the impact of early urbanization on the commoners during the Late Shang dynasty (ca. 1250–1046 B.C.). A total of 347 individuals examined in this study represent non-elites who were recovered from two different burial contexts (formally buried in lineage cemeteries and randomly scattered in refuse pits). Frequencies of enamel hypoplasia (childhood stress), *cribra orbitalia* (childhood stress and frailty) and osteoperiostitis (adult stress) were examined to assess systemic stress exposure. Our results reveal that there was no significant difference in the frequency of enamel hypoplasia between two burial groups and between sexes, suggesting these urban commoners experienced similar stresses during childhood, but significantly elevated levels of *cribra orbitalia* and osteoperiostitis were observed in the refuse pit female cohort. Theoretically, urbanization would have resulted in increased population density in the urban centre, declining sanitary conditions, and increased risk of resource shortage. Biologically, children would be more vulnerable to such physiological disturbance; as a result, high percentages of enamel hypoplasia (80.9% overall) and *cribra orbitalia* (30.3% overall) are observed in Yin commoners. Adults continued to suffer from stress, resulting in high frequencies of osteoperiostitis (40.0% total adults); in particular, in the refuse pit females who may also reflect a compound impact of gender inequality. Our data show that the non-elite urban population in the capital city of Late Shang Dynasty had experienced extensive stress exposure due to early urbanization with further social stratification only worsening the situation, and eventually contributing to collapse of the Shang Dynasty.

## Introduction

**Fig 1:**
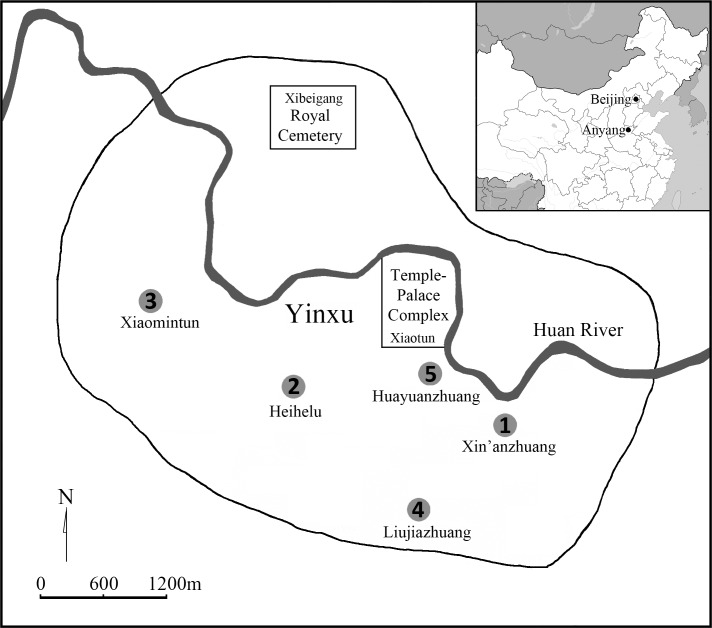
**Map of Yin and the locations of the sites: (1) Xin’anzhuang, (2) Xiaomintun, (3) Heihelu, (4) Liujiazhuang North, and (5) Huayuanzhuang East.**

Shang is a well-known early state-level society in Bronze Age China, and is the kingdom with the earliest written records of oracle bone inscriptions [1]. Its last capital, “Yin”, was unveiled in 1920s in the modern city of Anyang, Henan Province. Previous studies have shown that, following relocation of the Shang capital to Anyang, Yin rapidly expanded from a small residential area to a flourishing large urban centre (occupied ca. 1250–1046 B.C.) [2,3]. Centred around the palace-temple complex near a village called *Xiaotun* (小屯) and the royal cemetery at *Xibeigang* (西北岗) in the northwest, this ancient capital city had scattered over 35 sq. km across the Huan River valley in Anyang (114.19° E, 36.07° N) (Fig 1) [4].

The discovery of this Bronze Age mega-site “Yinxu (Ruins of Yin)” was one of the earliest scientific archaeological excavations conducted in China [5,6]. Numerous archaeological analyses indicate that the Yin-Shang Kingdom had managed to sustain a vast urban settlement. As population density dramatically increased, access to resources would have been significantly limited, exerting social, nutritional and pathogen-related stresses on the society [7–10]. Traditional archaeological works have relied heavily on written records and artifacts from elite contexts to understand Shang society. Without information about the commoners, our understanding of lifeways at Yinxu remains far from complete [11,12]. As a result, the study of non-elites may prove to be very useful in terms of characterizing and assessing impacts of early urbanization on changing society in ancient China.

This paper examines three systemic stress indicators (enamel hypoplasia, *cribra orbitalia*, and osteoperiostitis) on the human skeletal remains of non-elite Shang people recovered from two different burial contexts (lineage burials and refuse pits). The hypotheses tested are that: 1) physiological stress of urban inhabitants increased through time during the process of early urbanization of Yin; and 2) archaeologically-defined subgroups were differentially buffered against systemic stress in the urban community. The addition of this osteological dataset to archaeological and written evidence provides new insight into ancient Yin-Shang society.

## Archaeological Context

Yin is located on an alluvial plain on a tributary of the Yellow River in North China. At present the climate is continental-temperate with seasonal monsoon influences, and clearly distinct seasons [4]. As early as 8000BP, conditions in the Yellow River Valley supported dry-land millet-based agricultural practice [13,14]. By the Holocene Climate Optimum (7500 to 5000BP), pollen proxies and other paleoenvironmental evidence all indicate that North China was covered by broadleaf forest, suggesting a warmer and wetter climate than currently [15,16]. By the Late Shang (3000BP), climate had gradually turned cooler and dryer [17–20] initiating resource stress that continued through Yin-Shang and even into the Han and later dynasties. As climate deteriorated, resource stress required intensive deforestation for establishment of a multicropping agricultural system, seen archaeologically as a dramatic increase in the number of lithic tools for agriculture (such as knives and sickles) and supporting an amplified significance and reliance on agricultural practice [1,11,19,21–27]. With intensified social stratification and increased social division of labor, additional stressors were introduced and thus hastening health disparities among Yin inhabitants [1,7,18]. As supported by zooarchaeological and inscriptional evidence, animal husbandry was practiced on a grand scale during the Late Shang, not only for meat and raw materials for bone tool making, but more importantly as sacrifices in ritual activities to reinforce elite power and social identity [28–32]. Nonetheless, animal husbandry in Late Shang did not guarantee everyone in society to get equal amounts of animal protein. The differential access to adequate food resources, especially key nutrients, may have affected health and physiological development of people at Yin.

Given the relatively short occupation time of Yinxu, the diversity and scale of archaeological artifacts suggest that Yinxu had undergone continuous urban expansion with increasing population density [33]. This conclusion reflects recent studies of materials drawn from the many new archaeological sites excavated during the period of rapid economic growth in Anyang city over the past two decades. In addition to royal cemeteries, these new sites also include over 10,000 burials and numerous residential areas, refuse pits, storage pits, drainage systems, roads, workshops, and other features related to Shang people’s daily activities and lifeways. Based on oracle bone inscriptions and archaeological records, Song [34] once estimated that urban population size could have reached 450,000 by the end of Late Shang dynasty. Although the actual number of people is still under debate [35], Yin as a vast urban centre inevitably would encounter problems of human waste removal and difficulty of maintaining potable water as seen in other large settlements elsewhere in the world [36]. Therefore, given the high population density developing in the urban areas, gastrointestinal and other infections may be major stressors for the people at Yin.

Urbanization in Yinxu, a continuous and intensifying process, can be divided into four phases based on the typology of the unearthed artifacts, the stratigraphic correlation, and oracle bone inscription studies [37,38]. The trend towards decreasing diversity of the forms and styles of artifacts and architecture through time has been interpreted as indicating institutionalization and simplification during the process of early urbanization at Yin [39].

Yin-Shang people formed their own neighbourhoods within which the sphere of daily activities and interpersonal interactions including craft specialization, worship of common ancestors and burial in shaft pits in lineage cemeteries would take place [40,41]. Within each cemetery consistency in the pattern of burial goods, body position and orientation indicate their social, cultural and even genetic similarities [39,42]. Lineage cemeteries are thought to represent extended families/clans of Yin-Shang people [42] as suggested by substantial archaeological evidence. For example, a unique lineage emblem can be found on bronze vessels recovered from an individual cemetery [24,40,43]. In addition, the oracle bone inscriptions describe Yin burial practices that are consistent with what is observed archaeologically [44]. The English word “lineage” may not be the best choice to translate the Chinese character *zu* (族) meaning extended families/clans, in particular for bioarchaeological research, but it was adopted in the study to be consistent with other Shang archaeological studies.

Nonetheless human skeletal remains are frequently found outside these lineage cemeteries of Yinxu, in areas such as refuse pits located near or within the residential areas, sacrificial pits, house foundations, or wells [43,44]. In this study, human skeletons were recovered from layers of ash in residential areas without evidence of intentional burial, consistent body position, and often with no grave goods. Archaeological analyses of this type of burial are understudied in China although it has been found as early as in Neolithic Yellow River region [45–47]. Although it is difficult to determine social status of these skeletal individuals, this group of people is referred to in this study as the excluded cohort with no lineage connections [43,44], as those who were prevented from being buried in the normal lineage cemeteries.

Human skeletal remains are valuable and extremely informative, helping to illustrate the impacts of early urbanization on Yin-Shang society. They exhibit direct evidence of survivability and adaptation to changing social and cultural developments. Human skeletal materials, as living tissues, follow biological principles to response to myriad stresses during life. However, bone response can vary significantly with social and cultural experiences, thus creating a unique opportunity to study biocultural interactions in past human societies [48,49]. This is a significant and novel approach to the study of the early urbanization process in ancient China as there are few systematic studies focused on the health status and well-being of Shang people [50].

## Materials and Methods

### Materials

The sample evaluated in this study is composed of 347 skeletal individuals from five recently excavated archaeological sites in the Yinxu Conservation Area in Anyang city (Fig 1). These burials are dated to ca. 1250 B.C.– 1046 B.C., covering all four phases of Yinxu culture. The human skeletal remains were recovered from both lineage cemeteries and refuse pits in all four archaeological sites (Table 1) although the ratio of these two subgroups varies from one site to another.

**Table 1:** Skeletal samples used in this study (see S1 Table for detailed specimen numbers).

Site	Sample Size	Phase		Burial type	
		Early phase	Late phase	Lineage burials	Refuse pits
Xin’anzhuang*	178	38	48	162	16
Heihelu**	44	16	24	42	2
Xiaomintun [51]	87	1	54	76	11
Liujiazhuang North [52]	34	0	0	1	33
Huayuanzhuang East [53]	4	0	4	4	0
TOTAL	347	55	130	285	62

* The excavation of Xin’anzhuang site was led by the present study co-author Zhanwei Yue.

** The excavation of Heihelu site was led by the present study co-author Jigen Tang.

*, ** Site reports are not yet published.

Of the 285 individuals excavated from the lineage burials, 185 can be assigned to a clear chronological phase in Yinxu culture based on their archaeological contexts and typological traits of pottery. In order to increase the statistical power of analyses and facilitate temporal comparisons we combined Yinxu culture I and II to form early phase, and Yinxu culture III and IV to form late phase based on shared respective similarities of material culture [37,38].

Sixty-two individuals from refuse pits were examined in this study, unfortunately most of these individuals cannot be assigned to any chronological period due to the lack of associated cultural artifacts. As a result, these remains were excluded from temporal comparisons. However, refuse pit individuals were included in all other inter-subgroup comparisons.

### Methods

This osteoarchaeological research of excavated human skeletons of Late Shang dynasty (1250–1046 B.C.) was part of the collaborations “The Regional Archaeological Survey in the Huan River Valley” and “Human and Social Dynamics of Early Bronze Age China” between the Institute of Archaeology at the Chinese Academy of Social Sciences and the Department of Anthropology at the University of British Columbia. Dr. Zhichun Jing co-directed the joint project with Professor Jigen Tang from the Institute of Archaeology at the Chinese Academy of Social Sciences. The project had a formal permit issued by the China Bureau of Cultural Heritage Administration. The human skeletons analyzed were all archaeological samples that were excavated under supervision of Drs. Tang and Jing. All these samples are stored at the Anyang Work Station, a field facility of the Institute of Archaeology at the Chinese Academy of Social Science. The examination of human skeleton samples was conducted at the Anyang Work Station, strictly following the standards and protocols in accordance with the WMA Declaration of Helsinki–Ethical Principles for Medical Research Involving Human Subjects.

The preservation of the analyzed remains is generally good but the completeness of recovered skeletal elements varies considerably between individuals. For each individual, all bone surfaces were examined. Any morphology beyond the range of normal was documented. For the purpose of this study, only remains containing at least one of the following: frontal bone with at least one orbit, anterior teeth, and tibia were included in the analysis. As a result, the total number of individuals included in this study varies slightly for each of these three different stress indicators.

#### Age estimation and sex identification

Age estimations and sex identifications were made using the osteological methods in *the Standards* [54]. Adult age-at-death estimation was based on multiple techniques: pubic symphysis and auricular surface [55–57], cranial suture closure [58], and dental wear pattern [56,59]. Subadult age-at-death was determined using dental formation and eruption [60], epiphyseal closure [54] as well as diaphyseal length [61,62]. For more detailed analysis, individuals were grouped into six age categories [54] (Table 2). Adults with indeterminate age were excluded from statistical analyses.

**Table 2:** Age groups defined in this study.

Age group	Age range (Years)
1	Perinatal– 3
2	4–12
3	13–19
4	20–34
5	35–49
6	50 +

#### Indicators of systemic stress

**Fig 2:**
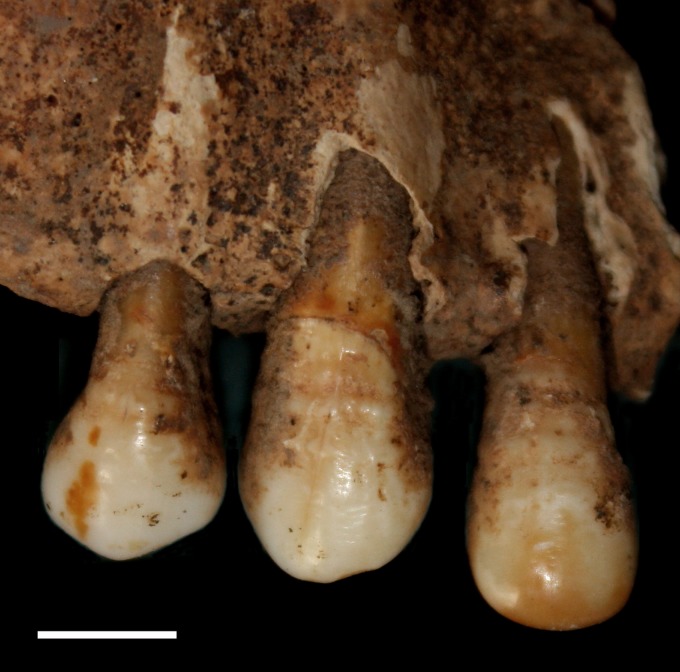
Linear enamel hypoplasia on the labial surface of right anterior maxillary dentition of 2003AXN192. Scale bar = 1cm.

Three indicators of systemic stress (linear enamel hypoplasia, *cribra orbitalia*, and osteoperiostitis) were evaluated macroscopically with the aid of a X10 eye loupe magnifier. Linear enamel hypoplasia (LEH), an indicator of physiological disruption and recovery during childhood, has been extensively used to explore the environmental impacts in humans, non-human primates and other species [63–68]. It is characterized as horizontal grooves or pits on the labial surface of both the deciduous and permanent anterior teeth (Fig 2). The cause of LEH is a systemic metabolic stress, it is multifactorial: maternal essential nutrient deficiencies, fever, starvation, congenital conditions, low birth weight and parasitic infection [69,70]. As the defects cannot be remodelled after the formation of enamel, LEH represents a chronological record of stress episodes that happened in the first 7 years of life (prenatally to 12 months for deciduous teeth, and birth to 7 years for permanent teeth) [71,72]. In this study, only maxillary anterior teeth (incisors and canines) and mandibular canines were documented, since these teeth are believed to be more sensitive to systemic stress than posterior teeth [71,73,74]. LEH was counted as present when one or more hypoplastic events were visible on any observed teeth.

**Fig 3:**
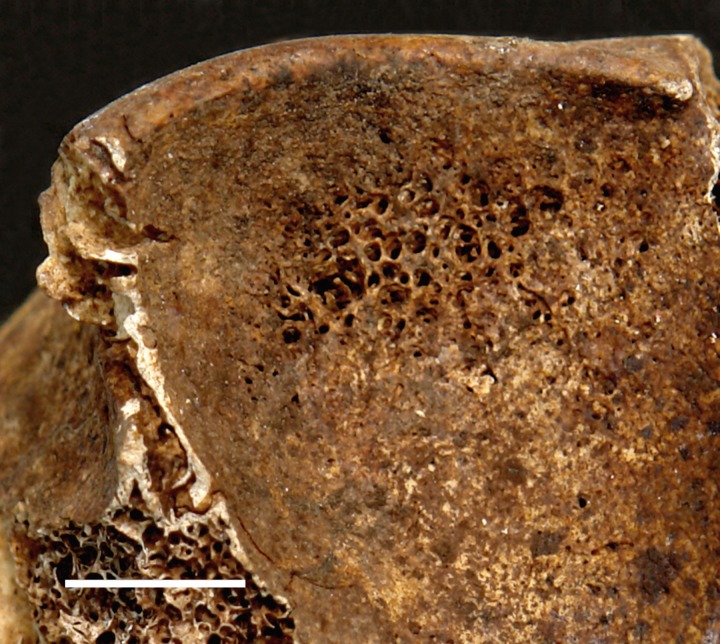
*Cribra orbitalia* on the right orbit of 2007AXAM30. Scale bar = 1cm.

*Cribra orbitalia* (CO) is porous sieve-like lesions on the superior orbital roof of the frontal bone (Fig 3). It is considered to be the earliest osseous expression of many types of anaemia and/or subperiosteal bleeding which can be caused and affected by multiple factors. Anaemias implicated include chronic childhood hemolytic, megaloblastic and/or iron deficiency. Multivitamin deficiencies (especially B_12_, B_6_ and/or C) or trauma, infection-related subperiosteal reaction can cause subperiosteal new bone formation. In addition, suboptimal osteoid calcification (rickets) and neoplastic processes may also present similar appearance [73–80]. Although the aetiology of CO is not fully understood [77,81], it is generally considered to be caused by synergistic reactions associated with increased pathogen load, gastrointestinal infestation, and/or malnutrition, prolonged breast-feeding, poor maternal health, and weaning diarrhea [36,77,82–89]. The condition was assessed if the individual has at least one orbit preserved for analysis. Status of the lesions was recorded as active at the time of death (unremodelled lesions with sharp pore margins) and healed at the time of death (remodelled lesions with rounded pore margins) [90]. Although porotic hyperostosis on the ectocranial vault surface appears similar to *cribra orbitalia*, only CO was included in this study due to the following: firstly, CO is more sensitive to stresses [91] and is more accurate for identifying than osteoporotic lesions on the vault, especially when the pitting is already healed; secondly, taphonomic changes on many skulls have damaged the bone surface, preventing reliable observation of porotic hyperostosis from being made.

**Fig 4:**
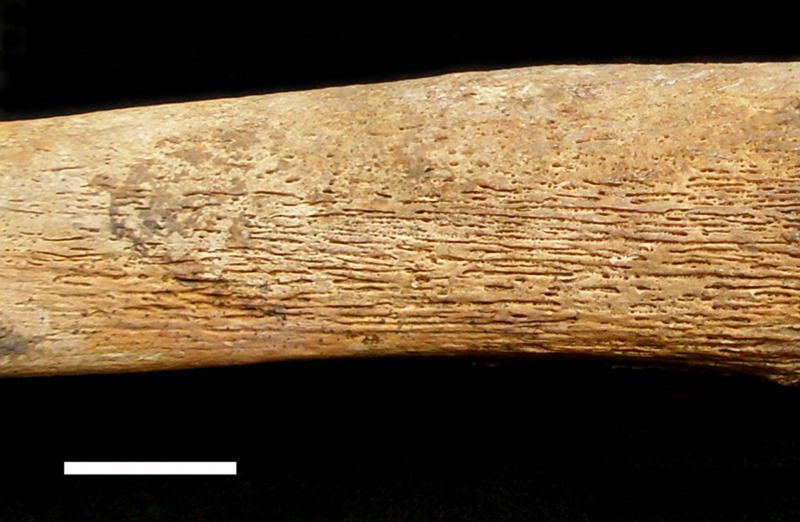
Osteoperiostitis on medial surface of right tibial shaft of 2007AXAM101. Scale bar = 2cm.

The third indicator, osteoperiostitis, is a layer of new woven bone deposited under an inflamed periosteum as the result of chronic inflammatory conditions and/or infection [92–94]. The lesion can be observed on the cortical surface primarily of major long bones (Fig 4). In this study, osteoperiostitis is used as a nonspecific indicator of physiological stress in response to endogenous or exogenous stressors in adulthood. Subadults were excluded from the statistical analysis since the sample size is too small to reveal meaningful pattern and the presence of periosteal lesions may sometimes be associated with normal growth [95]. The presence of subperiosteal new bone formation was recorded on the tibia as many previous studies have demonstrated that the tibia is the most affected skeletal element for this lesion [10,96–102]. All bone surfaces in every skeleton were examined for the presence and activity of periosteal lesions, following the criteria provided by Buikstra and Ubelaker [54]. As expected, the anterior surface of the tibia is the only bone /bone surface showing a much higher prevalence of the lesion while the other skeletal elements only reveal the lesion sporadically. Therefore, only the anterior surface of tibial diaphysis was included in the study for detailed analysis. Both left and right tibiae, if present, were examined for the presence of osteoperiostitis. Special care was made to distinguish the lesion from rough muscle attachments marks and localized trauma.

#### Statistical analysis

In this study, odd ratios (ORs) statistic was conducted to assess the differences between two groups of people (for example, males vs. females) to minimize the bias brought by non-identical age structures in the data [10, 103,104]. Following the analytical procedures described by Klaus and colleagues [104], ORs were calculated separately for each indicator in each defined age cohort. When the prevalence is higher in the first population compared (in this case, the males), OR is greater than1; if prevalence is higher in the second population compared (the females), OR is less than 1. For example, an OR of 2.82 would mean the prevalence of this indicator is 2.82 times greater in males; an OR of 0.78 would represent the prevalence is 1.28 times (1/0.78 = 1.28) greater in females.

A common odds ratio (OR_MH_) is then estimated and tested by Mantel-Haenszel statistic to determine the overall prevalence pattern between two groups of people as an age-related proportion. Significant differences between the samples in each comparison were determined by chi-square tests. Fisher’s exact tests were used when the cell number is less than 5. All statistical analyses were produced using SPSS 21. The detailed odds ratio values are presented in the supporting information section.

## Results

### Demographic profile

**Fig 5:**
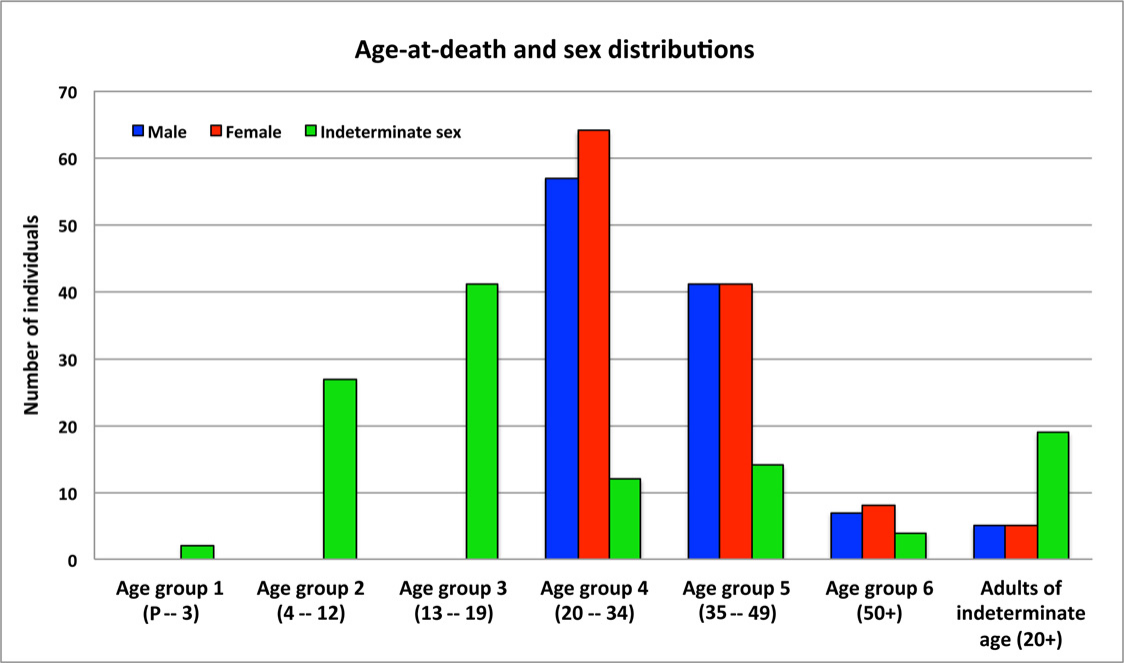
Age-at-death and sex distribution of the total sample. * * P = Perinatal.

The demographic profile of the sample was generated based on the human skeletal remains of 70 subadults and 277 adults (Fig 5): two infants (perinatal– 3 years), 27 children (4–12 years), and 41 adolescents (13–19 years), consisting 0.6%, 7.8%, and 11.8% of total individuals, respectively. The adult sample comprises 38.3% of total individuals aged 20 to 34 years (n = 133), 27.7% aged 35 to 49 years (n = 96), 5.5% aged over 50 years (n = 19), and 8.4% of adults (n = 29) with indeterminate age (older than 20 years). For adults, 39.7% are males (n = 110), 42.6% females (n = 118), and 17.6% individuals with indeterminate sex (n = 49).

When the sample was broken down by temporal phases (Table 3) and by two different burial aspects (lineage burials and refuse pits) (Table 4), the sex ratios do not show any significant difference by Kolmogorov-Smirnov test. However, the age distributions differ significantly between the two types of burials. The latter may also reflect sample bias since more lineage burials were included in the analysis.

**Table 3:** Age-at-death distribution of skeletons by sex and time period.*

	Subadults			Adults												Total
	Age group 1 (P– 3)	Age group 2 (4–12)	Age group 3 (13–19)	Age group 4 (20–34)			Age group 5 (35–49)			Age group 6 (50+)			Adults of indeterminate age (20+)			
				M	F	I	M	F	I	M	F	I	M	F	I	
Early phase	0	1	10	8	13	0	8	3	2	1	0	1	1	2	5	55
Late phase	0	8	11	17	18	5	23	21	7	4	5	3	2	1	5	130

* P = Perinatal; M = Male; F = Female; I = Adults of indeterminate sex.

**Table 4:** Age-at-death distribution of skeletons by sex and burial type.*

	Subadults			Adults												Total
	Age group 1 (P– 3)	Age group 2 (4–12)	Age group 3 (13–19)	Age group 4 (20–34)			Age group 5 (35–49)			Age group 6 (50+)			Adults of indeterminate age (20+)			
				M	F	I	M	F	I	M	F	I	M	F	I	
Lineage burials	2	19	33	41	51	11	38	36	14	6	7	4	4	3	16	285
Refuse pits	0	8	8	16	13	1	3	5	0	1	1	0	1	2	3	62

* P = Perinatal; M = Male; F = Female; I = Adults of indeterminate sex.

### Systemic stress indicators

The crude prevalence of LEH at Yin was found to be quite high across all age groups (Table 5). Of the 230 individuals with either permanent maxillary anterior teeth or mandibular canines preserved, 80.9% can be scored with presence of at least one LEH: 84.6% (n = 78) for males, 80.0% (n = 80) for females, and 80.8% (n = 52) for subadults (perinatal– 19 years).

**Table 5:** Crude prevalence data of systemic stress indicators by sex, temporal phase and burial type.*

			Enamel Hypoplasia		*Cribra Orbitalia*		Osteoperiostitis	
			N	%	N	%	N	%
Total			230	80.9	165	30.3	—	—
Subadults			52	80.8	23	52.2	—	—
Adults	Adults Total		178	80.9	142	26.8	180	40.0
	M		78	84.6	61	26.2	78	46.2
	F		80	80.0	69	27.5	83	36.1
Time Period	Early phase	M	12	83.3	6	33.3	12	33.3
		F	10	60.0	8	37.5	16	31.3
		Total	31	80.6	22	31.8	35	34.3
	Late phase	M	35	85.7	26	38.5	28	42.9
		F	32	78.1	27	25.9	25	24.0
		Total	92	81.5	65	33.8	57	31.6
Burial type	Lineage burials	M	67	85.1	50	30.0	63	46.0
		F	70	80.0	56	21.4	70	30.0
		Total	196	81.1	129	27.9	150	36.7
	Refuse pits	M	11	81.8	11	9.1	15	46.7
		F	10	80.0	13	53.8	13	69.2
		Total	34	79.4	36	38.9	29	58.6

*—Observations in adults only; N = total number of individuals observed; M = Male; F = Female.

Overall, of the 165 individuals with orbital roofs available for analysis, 30.3% exhibit evidence of *cribra orbitalia*: 26.2% (n = 61) for males, 27.5% (n = 69) for females; and 52.2% (n = 23) for subadults (perinatal– 19 years) (Table 5). Among all individuals, only 3 children (perinatal– 12 years) were found to have active *cribra orbitalia*, and all others exhibit remodelled lesions of *cribra orbitalia*.

Of the 180 adults with tibial diaphyses preserved, 40.0% were found to display osteoperiostitis: 46.2% (n = 78) for males and 36.1% (n = 83) for females (Table 5).

For adults, odds ratio tests of the overall comparison by sex reveal that females seem more likely to have an elevated prevalence of healed *cribra orbitalia*. In contrast males were more susceptible to enamel hypoplasia when they were young and to osteoperiostitis in adulthood than their female counterparts (Table 6; see detailed odds ratio results in S2 Table). However, the prevalences of these three stress indicators between sexes were not significantly different by chi-square analysis.

**Table 6:** Interpretation of odds ratio statistics for the overall comparison of systemic stress by sex, phase, and burial type.*

Pathological condition	Interpretation of odds ratio results		
	Male (M) vs. Female (F)	Early phase (E) vs. Late phase (L)	Lineage burials (B) vs. Refuse pits (R)
Enamel Hypoplasia	1.45 times M > F	1.19 times L > E	1.26 times B > R
*Cribra Orbitalia*	1.06 times F > M	1.59 times L > E	1.71 times R > B
Osteoperiostitis	1.61 times M > F	1.02 times L > E	2.43 times R > B*

* The difference is statistically significant (χ^2^_MH_ = 3.578, df = 1, P = 0.039).

#### Comparison by temporal phases

**Fig 6:**
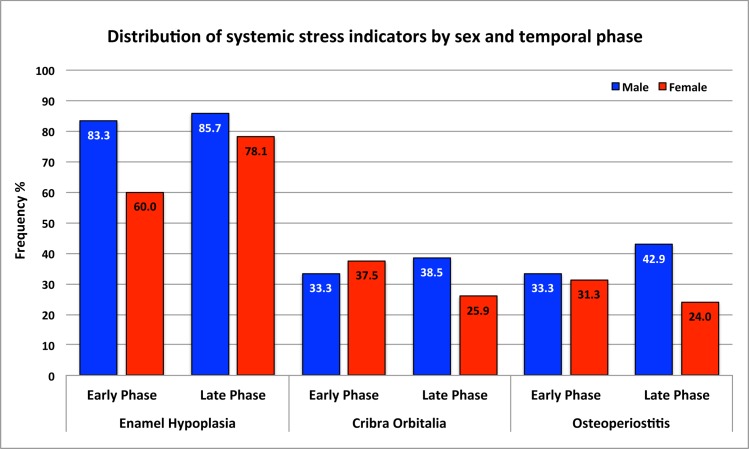
Crude prevalence comparisons of systemic stress indicators by sex and by temporal phase.

Odds ratio tests for the overall comparison are presented in Table 6 (see detailed odds ratio results in S3 Table). Enamel hypoplasia increased 1.19 times and *cribra orbitalia* prevalence is elevated 1.59 times in the late phase. People of the early phase were 1.02 times more likely to be affected by osteoperiostitis. However, no statistically significant temporal changes of the three systemic stress indicators have been detected. Crude prevalence comparisons are illustrated for the indicators by sex and by temporal phase (Fig 6; Table 5).

Within-phase comparisons by sex (Table 7; see detailed odds ratio results in S4 Table) reveal that males display a consistently higher prevalence of all of the stress indicators through time, however, the differences are not statistically significant.

**Table 7:** Interpretation of odds ratio results for the comparison of systemic stress by sex and by phase.

Pathological condition	Interpretation of odds ratio results	
	Within-phase comparisons	Between-phase comparisons
	Male (M) vs. Female (F)	Early phase (E) vs. Late phase (L)
	Early phase	Males
Enamel Hypoplasia	3.21 times M > F	1.15 times L > E
*Cribra Orbitalia*	3.33 times M > F	1.11 times L > E
Osteoperiostitis	1.53 times M > F	1.26 times L > E
	Late phase	Females
Enamel Hypoplasia	1.92 times M > F	4.29 times L > E
*Cribra Orbitalia*	1.67 times M > F	1.20 times E > L
Osteoperiostitis	2.59 times M > F	1.49 times E > L

Between-phase comparisons by sex (Table 7; see detailed odds ratio results in S5 Table) did not show any significantly elevated prevalence of the indicators in either males or females through time though males have slightly higher prevalences.

#### Comparison by burial type

**Fig 7:**
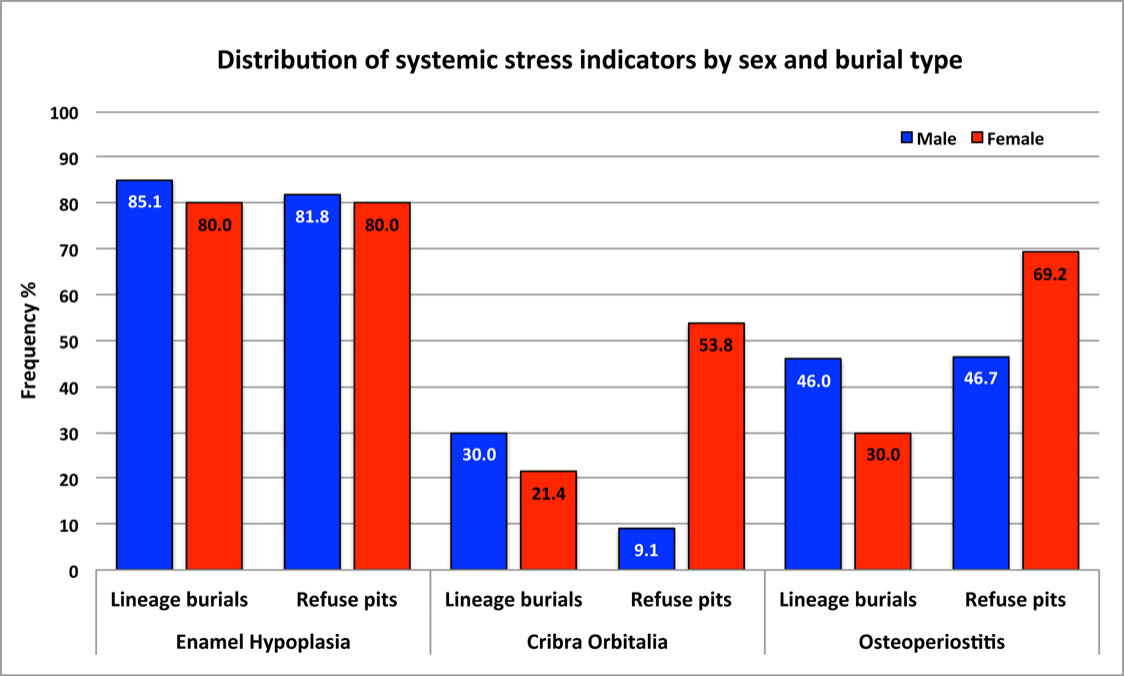
Crude prevalence comparisons of systemic stress indicators by sex and burial type.

The comparisons between the two types of burials reveal that the prevalence of osteoperiostitis significantly increased (2.43 times) in individuals from refuse pits (Table 6; see detailed odds ratio results in S6 Table). Additionally, individuals from refuse pits had nonsignificantly higher prevalence of *cribra orbitalia* (1.71 times) and lower prevalence of LEH (1.26 times) than those from lineage burials. Crude prevalence comparisons are illustrated for the indicators by sex and by burial type (Fig 7; Table 5).

Within-burial comparisons by sex (Table 8; see detailed odds ratio results in S7 Table) reveal that in lineage burials, males seem to have elevated prevalence of systemic stress indicators; specifically, males present significantly increased prevalence of osteoperiostitis (2.26 times). Within refuse pits, females show higher prevalence of *cribra orbitalia* (10 times) and osteoperiostitis (4.03 times) than the males, however, the differences are not statistically significant.

**Table 8:** Interpretation of odds ratio results for the comparison of systemic stress by sex and by burial type.

Pathological condition	Interpretation of odds ratio results	
	Within-burial comparisons	Between-burial type comparisons
	Male (M) vs. Female (F)	Lineage burials (B) vs. Refuse pits (R)
	Lineage burials	Males
Enamel Hypoplasia	1.56 times M > F	2.30 times B > R
*Cribra Orbitalia*	1.55 times M > F	3.53 times B > R
Osteoperiostitis	2.26 times M > F*	1.06 times B > R
	Refuse pits	Females
Enamel Hypoplasia	1.10 times M > F	1.04 times B > R
*Cribra Orbitalia*	10.00 times F > M	6.62 times R > B**
Osteoperiostitis	4.03 times F > M	7.69 times R > B***

* The difference is statistically significant (χ^2^_MH_ = 4.093, df = 1, P = 0.028).

** The difference is statistically significant (χ^2^_MH_ = 6.380, df = 1, P = 0.007).

*** The difference is statistically significant (χ^2^_MH_ = 7.249, df = 1, P = 0.006).

Between-burial type comparisons by sex (Table 8; see detailed odds ratio results in S8 Table) show that males from lineage burials have greater systemic stress indicator values than males from refuse pits, albeit not a statistically significant difference. Nevertheless, females of the refuse pits are affected more by *cribra orbitalia* and osteoperiostitis than females from lineage burials (6.62 times greater *cribra orbitalia* prevalence and 7.69 times greater osteoperiostitis prevalence).

## Discussion

The present study was undertaken to explore influences of early urbanization on health and well-being of Yin inhabitants in the Late Shang dynasty. Our data show that a considerable proportion of Yin inhabitants experienced a life with substantial physiological stress in childhood as evidenced by the high proportion of enamel hypoplasia (80.9%), suggesting systemic stress was pervasive across all sectors of Yin Shang society. However, following childhood some segments of society appear less buffered from further stress impacts than others. For example, females of refuse pits were significantly more affected by *cribra orbitalia* (CO) and osteoperiostitis than all the other groups. In addition, lineage cemetery males were more affected by osteoperiostitis than lineage cemetery females and refuse pits males. Contrary to our hypotheses that the three indicators would increase through time as urbanization progressed, there were no significant differences between the two phases.

There are at least three plausible explanations for these results. Perhaps the components of urbanization at Yin affecting inhabitants’ health had developed very rapidly following the establishment of Yin, were already in place in the preceding Shang society, or had been present since the transition to sedentary agricultural societies in the earlier Neolithic. In addition, the markers may not be the most sensitive indicators of the changes occurring during the rise of urban living. Furthermore, perhaps gender roles and social status differences in prevalence may better reflect the effects of urbanization on human health.

### Systemic stress of Yin inhabitants

Although the markers used in this study are considered to be non-specific indicators of stress, each marker reflects a different segment of a person’s life history. Enamel hypoplasia permanently documents in subadult and adult dentitions systemic stress episodes in childhood. Thus even though the Yin sample under-represents children the LEH prevalence presented here still provide substantial evidence of childhood physiological disruption. As *cribra orbitalia* (CO) is more frequently seen in children under five years of age, it is an additional indicator of childhood stress [82]. In adults the continued presence of CO suggests that healing and recovery were slowed or hindered and therefore its presence in adults provides a window on childhood frailty [82,105]. The use of *cribra orbitalia* in conjunction with LEH facilitates more comprehensive interpretations of the factors contributing to childhood non-specific stresses at Yin. The third marker osteoperiostitis (observed in this study in adults only) exhibits an adult record of systemic stress. Use of all three markers provides a more holistic view of lifeways of the Late Shang people at Yin with the potential to detect interactions between childhood stress exposure and risks of negative reactions to stressors later in life.

#### LEH

Enamel hypoplasia was high at Yin in all contexts (80.9%), which is higher than all earlier populations in ancient China as demonstrated in other studies for the Yellow River region ranging from 9% to 76% [106,107]. It should be pointed out that the frequency is assessed at the individual level but not at the tooth level (66.5% data not shown). The estimation of LEH prevalence in individuals often intrinsically exceeds the prevalence of the teeth, and hence it is important to compare data obtained from the same method quantifying lesion prevalence. However, because not all anterior teeth are present for observation in the individuals, reporting at the tooth may further underestimate LEH prevalence [108], making the figure less reliable. Therefore, the individual level prevalence of LEH is included for detailed analysis in this study.

High frequencies of LEH have been found in populations living in fluctuating marginal environments or experiencing subsistence transition. For instance, high prevalence of LEH (76.4%) was detected at the mixed agricultural/pastoral site of Houtaomuga in Northeast of China [109]. Similarly, LEH is high in ancient Japan: 48–65% for Jomon foragers and 63% for Yayoi agriculturalists [110]. Farther south in Vietnam and Thailand the prevalence in sedentary societies practicing agriculture is 71.7% at Da But and 67.3% at Metal period sites [111]. Additionally, a higher prevalence of LEH is also observed in a modern Chinese population (55.9%), when people are under the stress of famine [65].

Moreover, even higher prevalences have been observed in some complex societies from the Americas and Europe [96,112,113]. For example, Classic Maya populations from Xcambo, Mexico reached almost 100% (99.5%) [114], while the Villa El Salvador XII series from Peru exhibited 72.9% [112]. High prevalence continues in Medieval Europe in a population from Tirup, Denmark (86.3%) [115]. It is evident that high population density and urban life contribute to higher LEH prevalences.

Clinical studies show that enamel defects can be linked to numerous aetiological factors both hereditary and acquired through systemic and environmental conditions [72,108,116,117]. Taken together, this suggests that biologically no one in this society escaped stresses. The proximate causes of this non-specific systemic stress indicator such as vitamin deficiencies, limited food resources, quality and quantity of the food supply within the context of child-rearing practices and maternal diets, and heavy pathogen load in a sedentary urban lifestyle clearly can be expected during the urbanization process. As suggested, the high LEH frequency observed in this study reflects truly adverse impacts of early urbanization on all groups of Yin-Shang people, as seen through the lens of high vulnerability of children to physiological disturbances.

Since the majority of Yin people had experienced stress sufficient to halt enamel protein synthesis, trade-offs for early survival might be expected, for example survival of stress in childhood may compromise ability to survive future stress episodes [68,70]. However, the extremely high levels of LEH may obscure evidence of factor interactions and compromised future health [109]. The additional factors below are explored to tease out meaning from these results.

#### Cribra Orbitalia

Adult prevalence of *cribra orbitalia* at Yin is 26.8% (n = 142). From an extensive literature review, *cribra orbitalia* in adults appears to increase in prevalence through time and with each demographic transition [111,118–120]. In the Yellow River valley of China *cribra orbitalia* starts at earlier Neolithic sites (9000 to 5000BP) with values between zero and 25% [106,107](but also see [121]). During the later Neolithic (5000 to 4000BP) much higher frequencies have been recorded reaching up to 50% [106,107,122]. The trend towards elevated prevalence continues (44–56%) into dynastic China as population density and social complexity increase [106,107,123]. This trend is mirrored with low values in Neolithic Japan [110,124] and higher values (26.3 to 46%) in complex highly stratified societies in other regions of the world [89,106,125]. Clearly, Yin at the 26.8% seen in the present study is consistent with this trend.

However, the adult prevalence of *cribra orbitalia* tells only a partial story. Through partitioning of our sample by age into subadults and adults, biological and cultural factors contributing to stress exposure vulnerability and resilience can be further explored. The subadult prevalence of 52.2% (n = 23) is consistent with data reported in other studies. Over half of the subadults present the osseous lesion at Neolithic sites in China and Japan [110,122] and in Bronze Age China [123].

This seemingly contradictory outcome might be adequately interpreted through a biocultural approach. The higher value for subadults in our sample reflects the severe biological vulnerability of children. Although the adults are also exposed to the same levels of stress (they are living under the same conditions), they are biologically more resilient having mature immune systems and reduced requirements for growth and maintenance [126,127]. As a manifestation of bone response to stress, *cribra orbitalia* may not permanently document the stress episodes experienced. In addition, CO is more specific in causation. However, both markers reflect the same time frame of vulnerability of childhood. Taken together we might expect the lower prevalence of CO than LEH seen in this study. In adults remodelled orbital lesions may result from repeated childhood stress with slow recovery. These adults may have lacked sufficient buffering mechanisms, biological and/or cultural to allow for healing and eventual disappearance of the lesions. Thus CO may be a marker of use in both subadult and adult analyses.

In addition, adults are differentially buffered by cultural aspects of the Shang lineage system. Since Yin Shang is a highly stratified state level society [1,41,128], it can be speculated that the cultural buffering mechanisms for adults at Yin were unevenly distributed, as labour divisions and craft specialization were part of the social framework. Those with better buffering mechanisms may recover from the stress of their earlier life, and certainly reduce the chance of developing new lesions. The CO data in this study seem to fit nicely within this scenario. In adults, *cribra orbitalia* varies significantly when only female samples are compared (χ^2^_MH_ = 6.380, df = 1, P = 0.007). Refuse pit females exhibit 6.62 times greater CO (53.9%, n = 13) than lineage burial females (21.4%, n = 56) highlighting the influence of social and cultural factors on health indicator variables. If so, the CO data reveal not only biological response to stress itself but also to social and cultural influences on recovery and resilience.

#### Osteoperiostitis

As a non-specific indicator recording the baseline inferences of stress in adulthood, a large body of bioarchaeological scholarship links tibial osteoperiostitis with infectious agents such as *Staphylococcus* or *Streptococcus* [10,75]. However, other factors such as vitamin deficiencies [79,129,130] and repetitive trauma [93,97,131–134] have also been implicated. Therefore, its multifactorial aetiology often precludes specific interpretation of the lesion.

A high prevalence of osteoperiostitis in adults (40.0%, n = 180) was found in this study. However, in comparison to the values reported for six Neolithic sites in Central China (0.0%) [106], the forty per cent for Yin inhabitants seems remarkably elevated. An increase in prevalence has also been found in Southeast Asia with agricultural intensification from Neolithic to Early Bronze Age [135]. Similarly, studies in North America reveal a significant increase of osteoperiostitis frequency from precontact preagriculturalists to late contact populations [136] indicating a strong connection between this pathological condition and sedentary agricultural lifeways, and population density. European populations in more complex societies during the Late Antiquity–Early Middle Ages transition also exhibited primarily increases in osteoperiostitis prevalence [96,113].

As Yin was a craft production centre [29,41,137], Yin inhabitants lived and worked closely together, facilitating the transmission of acute or chronic systemic infections within the population. In addition, the lesions observed in Yin people may not just reflect the level of chronic systemic stress but also the impacts of malnutrition such as Vitamin B_3_ deficiency, rickets, scurvy, vascular disease, and traumatic injuries [75,97,129,130,134]. It is not surprising to see that the females in the refuse pits had suffered significantly more (7.69 times, χ^2^_MH_ = 7.249, df = 1, P = 0.006) than their female peers in lineage burials as the refuse pit females were less buffered by exclusion from the lineage system.

Recent studies show that tibial osteoperiostitis could also be related to inflammation caused by repetitive stress injuries [93,131,132,138,139]. When confronted by extensive chronic and repetitive strain that challenges the limits of bone elasticity, the periosteal-cortical bone interface of the tibia can be injured initiating new bone formation. The new bone serves to reduce further stress and potential fractures [93]. The observed alterations on the bone surface could be seen as a product of repetitive movements, resulting from heavy physical demand and/or extensive walking/running for long distances [131–133,138,139]. These scenarios have been linked to the elevated levels of subperiosteal inflammation seen among Andean women with heavy labour demands in Postcontact Peru [101].

Significantly higher prevalence of osteoperiostitis is found in lineage males than in lineage females (2.26 times, χ^2^_MH_ = 4.093, df = 1, P = 0.028). If the tibial osteoperiostitis is caused by repetitive physical demand as suggested above, our data may reflect the increasingly heavy workloads of lineage male craft specialists within the growing urban centre.

Although we could not rule out the possibility that some endemic infectious diseases might account for the high frequency of osteoperiostitis in the Yin population, there is so little periostitis observed on skeletal elements other than the tibia (data not shown) that no positive patterns to support their presence was observed. Therefore, the synergistic reaction of chronic infection with nutritional stress, high population density and repetitive physical stresses from extensive workloads of daily activities in the urban center were more likely to have contributed to the overall picture.

### Temporal trends in lineage burials

The three systemic stress indicators all show increases in prevalence from early to late phase, but odds ratio statistics failed to demonstrate any significant differences. This is not what we originally hypothesized: the prevalences were expected to increase drastically with intensifying urbanization at Yin. This discrepancy has many important implications.

Firstly, Yin may not be the first place where the early urbanization was initiated. Instead, the Shang King may have moved the last capital to Anyang with an established urban system in place and perhaps even with an urbanized population. New archaeological discoveries on the development of urbanism in the early states of Erlitou and Erligang in the Central Plain reveal abundant evidence of large urban centres well before the establishment of Yin, thus pointing to possible locations for initial urbanization [1].

Secondly, although other archaeological evidence of deteriorating environment and increasing population density through time [25,34,140] indicates the presence of intensifying sociocultural and political stresses, Yin society may have developed mechanisms to maintain the delicate balance between adaptation to changing social and environmental conditions and societal collapse. As supported by our data, within the 200 years of the regime, no dramatic increases in the prevalence of systemic stress indicators have been detected.

Thirdly, although Yin collapse was not instantaneous, our data of human remains failed to catch the expected increases in stress marker prevalence. This could be caused by: 1) small sample size; 2) sample bias towards the stable period of Yin; 3) the collapse happening too rapidly, leaving inadequate amounts of time for skeletons to develop any detectable lesions of the stress indicators used in this study.

Overall our data, showing no change through time, demonstrate the general patterns of stress exposure during the period of social balance at Yin. Further exploration of human skeletal remains in the initial and final stages of Yin occupation may generate new insights into the impact of urbanization on lifeways and health.

### Impact of early urbanization at Yin

Yin is considered to be the largest capital centre with early urbanization of its time in China. Regional deforestation, interregional trade networking, and internal social stratification that provided the resources needed to support the urban population and craft production have all been observed in the Yinxu archaeological record. Given the inferred poor living conditions at Yin, it is expected that the stress would be evident in the human skeletal remains.

Three analyzed physiological stress markers in this study show increases in prevalences when compared to earlier Neolithic societies in China, and also indicate trends similar to those seen in other societies elsewhere in the world. This confirms that urbanization exerts tremendous stress on the inhabitants. Furthermore, each of these three stress indicators has also revealed detailed and specific information about different segments of urban life history.

Without any possibility of tissue remodelling, LEH serves as a very sensitive marker to record childhood stress. With a prevalence of over 80%, our data is higher than any earlier Neolithic societies in China. Biologically, childhood is more vulnerable to stress, but socioculturally, childhood at Yin has not been effectively buffered against the impacts of stress. LEH clearly shows that the entire urban population (the commoners) at Yin was severely subjected to stresses.

With a chance of tissue remodelling, *cribra orbitalia* is used in this study to examine the body’s recovery and resilience from childhood stress. Osteoperiostitis assesses adult frailty. Our odds ratio results confirm that patterns of adult systemic stress in Late Shang dynasty of Yin were strongly conditioned by subtle social stratification.

In the context of lineage burials, health disparities between the two sexes were significantly different. Lineage males generally displayed a greater negative response to physiological stressors than their lineage female counterparts, indicating that lineage men may have been under greater physiological stress and have laboured more strenuously or intensively than the women. Heavy labour occupations of craft production such as bronze casting and tool making were within the professional realm of lineage males as seen by burial goods patterning [141]. For instance, bronze tools such as chisels and axes are found in male burials [141]. Additionally, one-sixth of the male burials contained bronze weapons, while weapons have been very rarely found in the same burial with spindle whorls that may represent female roles [40] (but see Parker Pearson [142] for a different interpretation of grave goods). This suggests that men predominated in the labour force and the military in Yin society. Although Yin commoners of lineage burials had stronger connections within certain lineages than those excluded individuals of refuse pits, they may have participated in more laborious though more prestigious activities such as craft production.

Concerning the refuse pit people, their social status is less clear, indicating the heterogeneous nature of this group in the city of Yin. Skeletons from refuse pits are usually interpreted as sacrificial victims [44] or slaves [46]. However, no osteological evidence has been identified for perimortem trauma such as decapitation or dismemberment cut marks that would clearly indicate human sacrifice. Life of the refuse pit people, especially females, appears to have been more stressful than for the lineage cohort, suggesting they belong to a marginalized and less privileged subgroup of Yin society. A recently published work on carbon and nitrogen isotopes from Xin’anzhuang site (which was also analyzed in this study) suggests that refuse pit people had lower animal protein intake in life that those of lineage burials [143]. Clearly, the process of early urbanization at Yin exacerbated the subtle health outcomes between the archaeologically-defined social subgroups.

## Conclusions

This study has examined systemic stresses that Yin inhabitants experienced during the Late Shang dynasty. This time period witnessed a dramatic increase in social stratification and urbanization, compounding their impacts on human health. Our study from two different burial contexts reveals important insights into the lifeways of the Shang people.

1. LEH (an indicator of childhood stress exposure) was pervasive throughout the entire sample. This indicates that substantial systemic stress, associated with increased population density and urbanization, affected the whole society. The universal vulnerability of children to physiological disturbances highlights the importance of biological factors of growth and development in the dental expression of childhood stress exposure.
2. *Cribra orbitalia* (an indicator of childhood stress and recovery and resilience in later life) was significantly higher in the female refuse pit cohort than all others, revealing differential impacts of stresses accompanying subtle social stratification on various groups within society.
3. The higher prevalence of osteoperiostitis (an indicator of adult stress exposure) was observed in the male lineage cohort than in the lineage females, inferring that men may have conducted higher levels of repetitive daily physical activities that affected the lower limbs than women. This is suggested to be related to increasing gender division of labour with early urbanization.

The observed patterns of variation show that the people at Yin must have experienced a stressful life caused by urbanization. Potentially, there were many adverse factors such as high population density, narrowed dietary choices, laborious lifestyle, and intensified social stratification that may have acted synergistically to affect the frailty of some individuals. However, gender roles and archaeologically-defined social groups differentiate Yin inhabitants in their susceptibility to adult systemic stress (as shown by *cribra orbitalia* and osteoperiostitis). This demonstrates the complexity of biocultural interactions in shaping human adaptation under conditions of stress.

The current study represents a new approach in Shang archaeology using human osteological evidence to explore the impacts of early urbanization on health and stress exposure of the Shang ‘masses’. Such insights from Shang commoners (rather than royal elites) will prove to be extremely useful for future synthetic analyses of skeletal remains and material culture, thus enhancing the existing archaeological narrative of Shang society.

## Supporting Information

S1 Table Skeletal samples used in this study. (DOCX)

S2 Table Odds ratio results for the overall comparison of systemic stress between males and females across age categories.* (DOCX)

S3 Table Odds ratio results for the comparison of systemic stress between early phase and late phase across age categories.* (DOCX)

S4 Table Odds ratio results for the comparison of systemic stress between males and females in early phase and late phase.* (DOCX)

S5 Table Odds ratio results for the comparison of systemic stress between early phase and late phase in males and females.* (DOCX)

S6 Table Odds ratio results for the overall comparison of systemic stress between different burial types.* (DOCX)

S7 Table Odds ratio results for the comparison of systemic stress between male and female inhabitants in burial types.* (DOCX)

S8 Table Odds ratio results for the comparison of systemic stress between different aspects of burial types in males and females.* (DOCX)
