# Supplementary material for: Osteoarchaeological Studies of Human Systemic Stress of Early Urbanization in Late Shang at Anyang, China
Source: PLoS One. 2016 Apr 6;11(4):e0151854. doi: 10.1371/journal.pone.0151854 (PMC4822842; doi:10.1371/journal.pone.0151854)
Supplement: S1 Table — (DOCX) [file pone.0151854.s001.docx]

S1 Table. Skeletal samples used in this study.

| **Sites** | **Specimen numbers** |
| --- | --- |
| **Xin’anzhuang** | 1. 2007AXAM10 |
|  | 1. 2007AXAM100 |
|  | 1. 2007AXAM101 |
|  | 1. 2007AXAM105 |
|  | 1. 2007AXAM106 |
|  | 1. 2007AXAM107 |
|  | 1. 2007AXAM108 |
|  | 1. 2007AXAM109 |
|  | 1. 2007AXAM111 |
|  | 1. 2007AXAM112 |
|  | 1. 2007AXAM113 |
|  | 1. 2007AXAM119 |
|  | 1. 2007AXAM120 |
|  | 1. 2007AXAM122 |
|  | 1. 2007AXAM123 |
|  | 1. 2007AXAM125 |
|  | 1. 2007AXAM126 |
|  | 1. 2007AXAM128 |
|  | 1. 2007AXAM131 |
|  | 1. 2007AXAM133 |
|  | 1. 2007AXAM134 |
|  | 1. 2007AXAM135 |
|  | 1. 2007AXAM136 |
|  | 1. 2007AXAM137 |
|  | 1. 2007AXAM138 |
|  | 1. 2007AXAM139 |
|  | 1. 2007AXAM140 |
|  | 1. 2007AXAM143 |
|  | 1. 2007AXAM144 |
|  | 1. 2007AXAM145 |
|  | 1. 2007AXAM146 |
|  | 1. 2007AXAM147 |
|  | 1. 2007AXAM148 |
|  | 1. 2007AXAM152 |
|  | 1. 2007AXAM154 |
|  | 1. 2007AXAM156 |
|  | 1. 2007AXAM157 |
|  | 1. 2007AXAM164 |
|  | 1. 2007AXAM165 |
|  | 1. 2007AXAM166 |
|  | 1. 2007AXAM168 |
|  | 1. 2007AXAM170 |
|  | 1. 2007AXAM173 |
|  | 1. 2007AXAM174 |
|  | 1. 2007AXAM175 |
|  | 1. 2007AXAM177 |
|  | 1. 2007AXAM178 |
|  | 1. 2007AXAM179 |
|  | 1. 2007AXAM18 |
|  | 1. 2007AXAM180 |
|  | 1. 2007AXAM181 |
|  | 1. 2007AXAM182 |
|  | 1. 2007AXAM183 |
|  | 1. 2007AXAM184 |
|  | 1. 2007AXAM185 |
|  | 1. 2007AXAM186 |
|  | 1. 2007AXAM187 |
|  | 1. 2007AXAM188 |
|  | 1. 2007AXAM190 |
|  | 1. 2007AXAM193 |
|  | 1. 2007AXAM194 |
|  | 1. 2007AXAM197 |
|  | 1. 2007AXAM198 |
|  | 1. 2007AXAM199 |
|  | 1. 2007AXAM200 |
|  | 1. 2007AXAM202 |
|  | 1. 2007AXAM203 |
|  | 1. 2007AXAM206 |
|  | 1. 2007AXAM208 |
|  | 1. 2007AXAM209 |
|  | 1. 2007AXAM21 |
|  | 1. 2007AXAM210 |
|  | 1. 2007AXAM212 |
|  | 1. 2007AXAM213 |
|  | 1. 2007AXAM215 |
|  | 1. 2007AXAM216 |
|  | 1. 2007AXAM221 |
|  | 1. 2007AXAM224 |
|  | 1. 2007AXAM227 |
|  | 1. 2007AXAM228 |
|  | 1. 2007AXAM229 |
|  | 1. 2007AXAM233 |
|  | 1. 2007AXAM234 |
|  | 1. 2007AXAM237 |
|  | 1. 2007AXAM238 |
|  | 1. 2007AXAM239 |
|  | 1. 2007AXAM240 |
|  | 1. 2007AXAM30 |
|  | 1. 2007AXAM32 |
|  | 1. 2007AXAM35 |
|  | 1. 2007AXAM36 |
|  | 1. 2007AXAM4 |
|  | 1. 2007AXAM40 |
|  | 1. 2007AXAM42 |
|  | 1. 2007AXAM43 |
|  | 1. 2007AXAM45 |
|  | 1. 2007AXAM46 |
|  | 1. 2007AXAM48 |
|  | 1. 2007AXAM49 |
|  | 1. 2007AXAM50 |
|  | 1. 2007AXAM52 |
|  | 1. 2007AXAM53 |
|  | 1. 2007AXAM54 |
|  | 1. 2007AXAM55 |
|  | 1. 2007AXAM56 |
|  | 1. 2007AXAM59 |
|  | 1. 2007AXAM6 |
|  | 1. 2007AXAM61 |
|  | 1. 2007AXAM64 |
|  | 1. 2007AXAM65 |
|  | 1. 2007AXAM66 |
|  | 1. 2007AXAM67 |
|  | 1. 2007AXAM69 |
|  | 1. 2007AXAM7 |
|  | 1. 2007AXAM70 |
|  | 1. 2007AXAM8 |
|  | 1. 2007AXAM81 |
|  | 1. 2007AXAM86 |
|  | 1. 2007AXAM9 |
|  | 1. 2007AXAM91 |
|  | 1. 2007AXAM92 |
|  | 1. 2007AXAM93 |
|  | 1. 2007AXAM94 |
|  | 1. 2007AXAM95 |
|  | 1. 2007AXAM99 |
|  | 1. 93AXAM112 |
|  | 1. 93AXAM116 |
|  | 1. 93AXAM124 |
|  | 1. 93AXAM126 |
|  | 1. 93AXAM130 |
|  | 1. 93AXAM138 |
|  | 1. 93AXAM140 |
|  | 1. 93AXAM142 |
|  | 1. 93AXAM153 |
|  | 1. 93AXAM154 |
|  | 1. 93AXAM155 |
|  | 1. 93AXAM157 |
|  | 1. 93AXAM168 |
|  | 1. 93AXAM188 |
|  | 1. 93AXAM2(M58) |
|  | 1. 93AXAM200 |
|  | 1. 93AXAM218 |
|  | 1. 93AXAM220 |
|  | 1. 93AXAM222 |
|  | 1. 93AXAM239 |
|  | 1. 93AXAM265 |
|  | 1. 93AXAM268 |
|  | 1. 93AXAM269 |
|  | 1. 93AXAM297 |
|  | 1. 93AXAM300 |
|  | 1. 93AXAM301 |
|  | 1. 93AXAM309 |
|  | 1. 93AXAM314 |
|  | 1. 93AXAM317 |
|  | 1. 93AXAM342 |
|  | 1. 93AXAM352 |
|  | 1. 93AXAM355 |
|  | 1. 93AXAM362 |
|  | 1. 93AXAM368 |
|  | 1. 93AXAM378 |
|  | 1. 93AXAM381 |
|  | 1. 93AXAM390 |
|  | 1. 93AXAM391 |
|  | 1. 2007AXAH102a |
|  | 1. 2007AXAG8 |
|  | 1. 2007AXAH102 |
|  | 1. 2007AXAH166 |
|  | 1. 2007AXAH178:3 |
|  | 1. 2007AXAH201 |
|  | 1. 2007AXAH214 |
|  | 1. 2007AXAH65 |
|  | 1. 2007AXAH77 |
|  | 1. 2007AXAM158 |
|  | 1. 2007AXAM218 |
|  | 1. 2007AXAM34 |
|  | 1. 93AXAM122 |
|  | 1. 93AXAM150 |
|  | 1. 93AXAM174 |
| **Heihelu** | 1. 96ABDM26 |
|  | 1. 96ABDM276 |
|  | 1. 97ABDM100 |
|  | 1. 97ABDM166 |
|  | 1. 97ABDM176 |
|  | 1. 97ABDM180 |
|  | 1. 97ABDM203 |
|  | 1. 97ABDM233 |
|  | 1. 97ABDM237 |
|  | 1. 97ABDM29 |
|  | 1. 97ABDM3 |
|  | 1. 97ABDM30 |
|  | 1. 97ABDM434 |
|  | 1. 97ABDM466 |
|  | 1. 97ABDM475 |
|  | 1. 97ABDM484 |
|  | 1. 97ABDM508 |
|  | 1. 97ABDM57 |
|  | 1. 97ABDM6 |
|  | 1. 97ABDM752 |
|  | 1. 97ABDM84 |
|  | 1. 97ABDM88 |
|  | 1. 98ABDM605 |
|  | 1. 98ABDM612 |
|  | 1. 98ABDM644 |
|  | 1. 98ABDM657 |
|  | 1. 98ABDM663 |
|  | 1. 98ABDM672 |
|  | 1. 98ABDM693 |
|  | 1. 98ABDM701 |
|  | 1. 98ABDM704 |
|  | 1. 98ABDM705 |
|  | 1. 98ABDM710 |
|  | 1. 98ABDM718 |
|  | 1. 98ABDM742 |
|  | 1. 98ABDH1:2 |
|  | 1. 99ABDM623 |
|  | 1. 99ABDM792 |
|  | 1. 99ABDT2H1:3 |
|  | 1. 99ABDT2H2:2 |
|  | 1. 99ABDT2H9:5 |
|  | 1. 97ABDT2:5 |
|  | 1. 97ABDT2:9 |
|  | 1. 99ABDT2H1:3 |
| **Xiaomintun** | 1. 2003AXNM114 |
|  | 1. 2003AXNM140 |
|  | 1. 2003AXNM161 |
|  | 1. 2003AXNM163 |
|  | 1. 2003AXNM171 |
|  | 1. 2003AXNM177 |
|  | 1. 2003AXNM184 |
|  | 1. 2003AXNM191 |
|  | 1. 2003AXNM192 |
|  | 1. 2003AXSM107 |
|  | 1. 2003AXSM204 |
|  | 1. 2003AXSM219 |
|  | 1. 2003AXSM225 |
|  | 1. 2003AXSM230 |
|  | 1. 2003AXSM237 |
|  | 1. 2003AXSM247 |
|  | 1. 2003AXSM354 |
|  | 1. 2003AXSM358 |
|  | 1. 2003AXSM360 |
|  | 1. 2003AXSM377 |
|  | 1. 2003AXSM378 |
|  | 1. 2003AXSM385 |
|  | 1. 2003AXSM386 |
|  | 1. 2003AXSM389 |
|  | 1. 2003AXSM390 |
|  | 1. 2003AXSM399 |
|  | 1. 2003AXSM422 |
|  | 1. 2003AXSM46 |
|  | 1. 2003AXSM556 |
|  | 1. 2003AXSM566 |
|  | 1. 2003AXSM580 |
|  | 1. 2003AXSM591 |
|  | 1. 2003AXSM60 |
|  | 1. 2003AXSM625 |
|  | 1. 2003AXSM635 |
|  | 1. 2003AXSM636 |
|  | 1. 2003AXSM644 |
|  | 1. 2003AXSM657 |
|  | 1. 2003AXSM666 |
|  | 1. 2003AXSM667 |
|  | 1. 2003AXSM689 |
|  | 1. 2003AXSM727 |
|  | 1. 2003AXSM732 |
|  | 1. 2003AXSM734 |
|  | 1. 2003AXSM736 |
|  | 1. 2003AXSM740 |
|  | 1. 2003AXSM748 |
|  | 1. 2003AXSM752 |
|  | 1. 2003AXSM756 |
|  | 1. 2003AXSM758 |
|  | 1. 2003AXSM761 |
|  | 1. 2003AXSM764 |
|  | 1. 2003AXSM766 |
|  | 1. 2003AXSM769 |
|  | 1. 2003AXSM778 |
|  | 1. 2003AXSM841 |
|  | 1. 2003AXSM848 |
|  | 1. 2003AXSM859 |
|  | 1. 2003AXSM862 |
|  | 1. 2003AXSM877 |
|  | 1. 2003AXSM907 |
|  | 1. 2003AXSM910 |
|  | 1. 2004AXSM922 |
|  | 1. 2004AXSM928 |
|  | 1. 2004AXSM933 |
|  | 1. 2004AXSM938 |
|  | 1. 2004AXSM940 |
|  | 1. 2004AXSM941 |
|  | 1. 2004AXSM943 |
|  | 1. 2004AXSM944 |
|  | 1. 2004AXSM949 |
|  | 1. 2004AXSM964 |
|  | 1. 2004AXSM967 |
|  | 1. 2004AXSM979 |
|  | 1. 2004AXSM980 |
|  | 1. 2004AXSM982 |
|  | 1. 2003AXNH56(M176) |
|  | 1. 2003AXNM182 |
|  | 1. 2003AXNM186 |
|  | 1. 2003AXNM190 |
|  | 1. 2003AXSH226:4(M968) |
|  | 1. 2003AXSH226:4(M973) |
|  | 1. 2003AXSH226:6(M972) |
|  | 1. 2003AXSH288:7 |
|  | 1. 2003AXSH493 |
|  | 1. 2003AXSM662 |
|  | 1. 2004AXSM976 |
| **Liujiangzhuang North** | 1. 2011ALNM381 |
|  | 1. 2008ALNH134:4 |
|  | 1. 2008ALNH197:012 |
|  | 1. 2008ALNH223:8 |
|  | 1. 2008ALNH245 |
|  | 1. 2008ALNH265:A |
|  | 1. 2008ALNH265:B |
|  | 1. 2008ALNH279 |
|  | 1. 2008ALNH350 |
|  | 1. 2008ALNH351:A |
|  | 1. 2008ALNH351:B |
|  | 1. 2008ALNH351:C |
|  | 1. 2008ALNH524:014 |
|  | 1. 2008ALNH524:015 |
|  | 1. 2008ALNH524:016 |
|  | 1. 2008ALNH524:017 |
|  | 1. 2008ALNH524:051 |
|  | 1. 2008ALNH527:018 |
|  | 1. 2008ALNH527:019 |
|  | 1. 2008ALNH527:020 |
|  | 1. 2008ALNH527:023 |
|  | 1. 2008ALNH527:024 |
|  | 1. 2008ALNH527:025 |
|  | 1. 2008ALNH527:053 |
|  | 1. 2008ALNH570:04 |
|  | 1. 2008ALNH586:034 |
|  | 1. 2008ALNH587:027 |
|  | 1. 2008ALNH587:028 |
|  | 1. 2008ALNH587:029 |
|  | 1. 2008ALNH587:030 |
|  | 1. 2008ALNH587:031 |
|  | 1. 2008ALNH587:032 |
|  | 1. 2008ALNH587:033 |
|  | 1. 2008ALNH587:055 |
| **Huayuanzhuang East** | 1. 2001HDM65 |
|  | 1. 2002HDM77 |
|  | 1. 2002HDM84 |
|  | 1. 2002HDM90 |
