# Supplementary material for: Osteoarchaeological Studies of Human Systemic Stress of Early Urbanization in Late Shang at Anyang, China
Source: PLoS One. 2016 Apr 6;11(4):e0151854. doi: 10.1371/journal.pone.0151854 (PMC4822842; doi:10.1371/journal.pone.0151854)
Supplement: S2 Table — (DOCX) [file pone.0151854.s002.docx]

S2 Table. Odds ratio results for the overall comparison of systemic stress between males and females across age categories.*

| Pathological condition | OR_4_^a^ | OR_5_ | OR_6_ | OR_MH_^b^ | Interpretation |
| --- | --- | --- | --- | --- | --- |
| Enamel Hypoplasia | 2.82 | 0.81 | — | 1.45 | 1.45 times greater prevalence in males |
| *Cribra Orbitalia* | 0.78 | 1.54 | 0.33 | 0.93 | 1.06 times greater prevalence in females |
| Osteoperiostitis | 1.57 | 1.13 | 20.00 | 1.61 | 1.61 times greater prevalence in males |

* — ORs were not calculated when any cell values are zero.

^a^ OR_4_ to OR_6_ correspond to individual odds ratios for adult age groups 4 to 6 (see Table 2) .

^b^ OR_MH_, the Mantel-Haenszel common odds ratio of each pathological condition.
