# Supplementary material for: Osteoarchaeological Studies of Human Systemic Stress of Early Urbanization in Late Shang at Anyang, China
Source: PLoS One. 2016 Apr 6;11(4):e0151854. doi: 10.1371/journal.pone.0151854 (PMC4822842; doi:10.1371/journal.pone.0151854)
Supplement: S3 Table — (DOCX) [file pone.0151854.s003.docx]

S3 Table. Odds ratio results for the comparison of systemic stress between early phase and late phase across age categories.*

| Pathological condition | OR_1_^a^ | OR_2_ | OR_3_ | OR_4_ | OR_5_ | OR_6_ | OR_MH_^b^ | Interpretation |
| --- | --- | --- | --- | --- | --- | --- | --- | --- |
| Enamel Hypoplasia | — | — | — | 0.27 | 1.80 | — | 0.84 | 1.19 times greater prevalence in the Late phase |
| *Cribra Orbitalia* | — | — | — | 1.94 | — | — | 0.63 | 1.59 times greater prevalence in the Late phase |
| Osteoperiostitis |  |  |  | 1.17 | 1.33 | — | 0.98 | 1.02 times greater prevalence in the Late phase |

* — ORs were not calculated when any cell values are zero. Blank area, observations in adults only.

^a^ OR_1_ to OR_6_ correspond to individual odds ratios for age groups 1 to 6 (see Table 2).

^b^ OR_MH_, the Mantel-Haenszel common odds ratio of each pathological condition.
