# Supplementary material for: Osteoarchaeological Studies of Human Systemic Stress of Early Urbanization in Late Shang at Anyang, China
Source: PLoS One. 2016 Apr 6;11(4):e0151854. doi: 10.1371/journal.pone.0151854 (PMC4822842; doi:10.1371/journal.pone.0151854)
Supplement: S4 Table — (DOCX) [file pone.0151854.s004.docx]

S4 Table. Odds ratio results for the comparison of systemic stress between males and females in early phase and late phase.*

| Pathological condition | OR_4_^a^ | OR_5_ | OR_6_ | OR_MH_^b^ | Interpretation |
| --- | --- | --- | --- | --- | --- |
| Early phase |  |  |  |  |  |
| Enamel Hypoplasia | 2.40 | 6.00 | — | 3.21 | 3.21 times greater prevalence in males |
| *Cribra Orbitalia* | 3.33 | — | — | 3.33 | 3.33 times greater prevalence in males |
| Osteoperiostitis | 3.00 | 0.25 | — | 1.53 | 1.53 times greater prevalence in males |
| Late phase |  |  |  |  |  |
| Enamel Hypoplasia | — | 1.36 | — | 1.92 | 1.92 times greater prevalence in males |
| *Cribra Orbitalia* | 0.57 | 5.57 | 0.50 | 1.67 | 1.67 times greater prevalence in males |
| Osteoperiostitis | 2.00 | 2.29 | 8.00 | 2.59 | 2.59 times greater prevalence in males |

* — ORs were not calculated when any cell values are zero.

^a^ OR_4_ to OR_6_ correspond to individual odds ratios for adult age groups 4 to 6 (see Table 2).

^b^ OR_MH_, the Mantel-Haenszel common odds ratio of each pathological condition.
