# Supplementary material for: Osteoarchaeological Studies of Human Systemic Stress of Early Urbanization in Late Shang at Anyang, China
Source: PLoS One. 2016 Apr 6;11(4):e0151854. doi: 10.1371/journal.pone.0151854 (PMC4822842; doi:10.1371/journal.pone.0151854)
Supplement: S5 Table — (DOCX) [file pone.0151854.s005.docx]

S5 Table. Odds ratio results for the comparison of systemic stress between early phase and late phase in males and females.*

| Pathological condition | OR_4_^a^ | OR_5_ | OR_6_ | OR_MH_^b^ | Interpretation |
| --- | --- | --- | --- | --- | --- |
| Males |  |  |  |  |  |
| Enamel Hypoplasia | — | 2.00 | — | 0.87 | 1.15 times greater prevalence in the Late phase |
| *Cribra Orbitalia* | 7.00 | — | — | 0.90 | 1.11 times greater prevalence in the Late phase |
| Osteoperiostitis | 1.75 | 0.29 | — | 0.80 | 1.26 times greater prevalence in the Late phase |
| Females |  |  |  |  |  |
| Enamel Hypoplasia | 0.15 | 0.46 | — | 0.23 | 4.29 times greater prevalence in the Late phase |
| *Cribra Orbitalia* | 1.20 | — | — | 1.20 | 1.20 times greater prevalence in the Early phase |
| Osteoperiostitis | 1.17 | 2.67 | — | 1.49 | 1.49 times greater prevalence in the Early phase |

* — ORs were not calculated when any cell values are zero.

^a^ OR_4_ to OR_6_ correspond to individual odds ratios for adult age groups 4 to 6 (see Table 2).

^b^ OR_MH_, the Mantel-Haenszel common odds ratio of each pathological condition.
