# Supplementary material for: Osteoarchaeological Studies of Human Systemic Stress of Early Urbanization in Late Shang at Anyang, China
Source: PLoS One. 2016 Apr 6;11(4):e0151854. doi: 10.1371/journal.pone.0151854 (PMC4822842; doi:10.1371/journal.pone.0151854)
Supplement: S6 Table — (DOCX) [file pone.0151854.s006.docx]

S6 Table. Odds ratio results for the overall comparison of systemic stress between different burial types.*

| Pathological condition | OR_1_^a^ | OR_2_ | OR_3_ | OR_4_ | OR_5_ | OR_6_ | OR_MH_^b^ | Interpretation |
| --- | --- | --- | --- | --- | --- | --- | --- | --- |
| Enamel Hypoplasia | — | 0.55 | 4.20 | 1.39 | 0.74 | — | 1.26 | 1.26 times greater prevalence in lineage burials |
| *Cribra Orbitalia* | — | 2.25 | 0.27 | 0.95 | 0.11 | — | 0.58 | 1.71 times greater prevalence in refuse pits |
| Osteoperiostitis |  |  |  | 0.55 | 0.12 | 0.71 | **0.41^c^** | **2.43 times greater prevalence in refuse pits** |

* — ORs were not calculated when any cell values are zero. Blank area, observations in adults only.

^a^ OR_1_ to OR_6_ correspond to individual odds ratios for age groups 1 to 6.

^b^ OR_MH_, the Mantel-Haenszel common odds ratio of each pathological condition.

^c^ The difference is statistically significant (χ^2^ = 3.578, df = 1, P = 0.039).
