# Supplementary material for: Osteoarchaeological Studies of Human Systemic Stress of Early Urbanization in Late Shang at Anyang, China
Source: PLoS One. 2016 Apr 6;11(4):e0151854. doi: 10.1371/journal.pone.0151854 (PMC4822842; doi:10.1371/journal.pone.0151854)
Supplement: S7 Table — (DOCX) [file pone.0151854.s007.docx]

S7 Table. Odds ratio results for the comparison of systemic stress between male and female inhabitants in burial types.*

| Pathological condition | OR_4_^a^ | OR_5_ | OR_6_ | OR_MH_^b^ | Interpretation |
| --- | --- | --- | --- | --- | --- |
| Lineage burials |  |  |  |  |  |
| Enamel Hypoplasia | 2.90 | 1.00 | — | 1.56 | 1.56 times greater prevalence in males |
| *Cribra Orbitalia* | 1.62 | 2.56 | 0.33 | 1.55 | 1.55 times greater prevalence in males |
| Osteoperiostitis | 2.17 | 1.71 | 16.00 | **2.26^c^** | **2.26 times greater prevalence in males** |
| Refuse pits |  |  |  |  |  |
| Enamel Hypoplasia | 3.20 | — | — | 1.10 | 1.10 times greater prevalence in males |
| *Cribra Orbitalia* | 0.10 | — | — | 0.10 | 10.00 times greater prevalence in females |
| Osteoperiostitis | 0.34 | — | — | 0.25 | 4.03 times greater prevalence in females |

* — ORs were not calculated when any cell values are zero.

^a^ OR_4_ to OR_6_ correspond to individual odds ratios for adult age groups 4 to 6 (see Table 2).

^b^ OR_MH_, the Mantel-Haenszel common odds ratio of each pathological condition.

^c^ The difference is statistically significant (χ^2^ = 4.093, df = 1, P = 0.028).
