# Supplementary material for: Osteoarchaeological Studies of Human Systemic Stress of Early Urbanization in Late Shang at Anyang, China
Source: PLoS One. 2016 Apr 6;11(4):e0151854. doi: 10.1371/journal.pone.0151854 (PMC4822842; doi:10.1371/journal.pone.0151854)
Supplement: S8 Table — (DOCX) [file pone.0151854.s008.docx]

S8 Table. Odds ratio results for the comparison of systemic stress between different aspects of burial types in males and females.*

| Pathological condition | OR_4_^a^ | OR_5_ | OR_6_ | OR_MH_^b^ | Interpretation |
| --- | --- | --- | --- | --- | --- |
| Males |  |  |  |  |  |
| Enamel Hypoplasia | 1.88 | 3.00 | — | 2.30 | 2.30 times greater prevalence in the lineage burials |
| *Cribra Orbitalia* | 3.53 | — | — | 3.53 | 3.53 times greater prevalence in the lineage burials |
| Osteoperiostitis | 1.17 | 0.67 | — | 1.06 | 1.06 times greater prevalence in the lineage burials |
| Females |  |  |  |  |  |
| Enamel Hypoplasia | 2.07 | — | — | 1.04 | 1.04 times greater prevalence in the lineage burials |
| *Cribra Orbitalia* | 0.22 | **0.07^e^** | — | **0.15^c^** | **6.62 times greater prevalence in the refuse pits** |
| Osteoperiostitis | 0.19 | — | — | **0.13^d^** | **7.69 times greater prevalence in the refuse pits** |

* — ORs were not calculated when any cell values are zero.

^a^ OR_4_ to OR_6_ correspond to individual odds ratios for adult age groups 4 to 6 (see Table 2).

^b^ OR_MH_, the Mantel-Haenszel common odds ratio of each pathological condition.

^c^ The difference is statistically significant (χ^2^ = 6.380, df = 1, P = 0.007).

^d^ The difference is statistically significant (χ^2^ = 7.249, df = 1, P = 0.006).

^e^ The prevalence is about 14.29 times greater in the refuse pit females at age 35 to 49 years. The difference is statistically significant (χ^2^ = 5.888, df = 1, P = 0.042).
